# Parents’ Experiences Regarding School Meals during the COVID-19 Pandemic

**DOI:** 10.3390/nu13124211

**Published:** 2021-11-24

**Authors:** Ilze Beitane, Sandra Iriste, Rita Riekstina-Dolge, Gita Krumina-Zemture, Marta Eglite

**Affiliations:** Department of Nutrition, Faculty of Food Technology, Latvia University of Life Sciences and Technologies, LV-3004 Jelgava, Latvia; sandra.iriste@llu.lv (S.I.); rita.riekstina@llu.lv (R.R.-D.); gita.krumina@llu.lv (G.K.-Z.); marta.eglite@llu.lv (M.E.)

**Keywords:** school meals, catering allowance, food packs, parent, pandemic

## Abstract

The onset of the COVID-19 pandemic required not only the reorientation of learning to remote form but also a change in the form of state-funded school lunches. One of the forms of school catering allowance was food packs, which obligated parents to prepare a warm lunch for the pupil from products included in food packs. As the responsibility for providing a warm lunch for the pupil was transferred to the parents, it was important to understand the parents’ experience. The survey was used to gather parents’ experiences of school catering allowance received during the pandemic using survey administration software—Google forms; 5166 respondents from different regions of Latvia took part in the survey. The school catering allowance in the form of food packs (83.7%) can be considered successful as over 70% of respondents rated it as positive, giving a rating of 7 (good) or above. Parents from Vidzeme and Latgale had the most positive experience with food packs. The parents appreciated the support they received, stating that it provided a certain sense of security during the crisis. Parental dissatisfaction was related to the composition of food packs, lack of local products and unacceptable products, such as canned meat and fish.

## 1. Introduction

Parental involvement and responsibility play an essential role in developing and strengthening a child’s eating habits, as well as in determining the body weight status [1]. In addition to parental contributions, an important component in the provision of nutrition, are school meals, which are valued for higher nutrient quality than out-of-school meals [2]. The impact of nutrition from school meals diminished at the beginning of the COVID-19 pandemic and then increased due to pupils being provided with food packs for lunch. The evaluation of the nutritional and energy value of Latvian school food packs has been provided in the previous study [3], however, a topical issue is the parents’ experience and evaluation of the received food packs, and whether the necessary support was provided during the pandemic. The goal of school meals (during the pandemic—food packs) is to provide pupils with a healthy meal and to develop healthy eating habits. During the pandemic, the country had various restrictions that promoted a sedentary lifestyle, pupils were forced to spend a lot of time on smart devices studying, communicating with friends and relatives, which in turn is one of the conditions for the development of obesity among children [4]; in such a situation, dietary choices become essential. Various studies have shown that the pandemic restrictions have had a negative effect on children’s and adolescents’ eating habits, making them less healthy [5,6].

According to the official statistics of Latvia, 685 schools started the school year in 2020; there were 179,882 pupils in grades 1 to 9 and 36,091 pupils in grades 10 to 12 [7]. Grade 1 to 4 pupils in Latvia are provided with free lunches from the state and municipalities budget, where the minimum cost per lunch and one pupil is EUR 1.42. This does not exclude the possibility for local municipalities to provide additional funding by increasing the cost per meal for one pupil or by increasing the number of pupils receiving free lunches. In 2020, the state budget funding for the provision of free lunches for grades 1 to 4 amounted to EUR 6,357,594 [8].

During the pandemic, pupils’ learning was reoriented to a remote form, which also required changes in the provision of free lunches. One of the options was to provide families of pupils with food packs for preparing a warm lunch at home. Guidelines on the composition of food packs were set by the Ministry of Health [9], considering the nutritional and energy needs of pupils at lunch. Food packs prepared by municipalities/schools can be assessed in two ways; on the one hand, it provided assistance to families, but on the other hand, it obligated the family to prepare lunch for the pupil. Here, it is important to understand how families perceived this help, whether it was seen as providing great support, whether the current composition of food packs met the family’s expectations, whether it provided pupils with healthy, warm lunches, and so on. The aim of the study was to analyse the parents’ experience regarding the provision of free lunches for pupils during the pandemic when teaching was performed remotely.

Food packs were offered by the government of the Republic of Latvia/municipalities to assist pupils’ families during the COVID-19 pandemic and remote learning. This research is unique because (1) it took place in the Republic of Latvia (a regional focus), (2) it took place during the COVID-19 pandemic; therefore, comparative studies with the experience of other countries could not be carried out.

## 2. Materials and Methods

An online questionnaire, using the survey administration software—Google forms, was sent to all Latvian schools that offer teaching from grades 1 to 4, with a request to send them to parents to fill them out. The questionnaire consisted of 45 questions in eight sections. Five questions were closed type, two questions were open type, four were line scale questions, and the rest were partially open questions. This article presents and analyzes the obtained results. The analyzed survey questions were divided into three groups: (1) school catering allowance received by the family for pupils; (2) composition of food packs, their use and parental opinion; (3) family characteristics.

The pilot study was conducted with 20 parents, approbating the questionnaire. The results of the pilot study showed that all the questions in the questionnaire were clear and understandable. None of the questions required further clarification.

A total of 5166 respondents participated in the survey, which took place from April to June 2021. Of the 5166 respondents, 4324 received food packs and were able to complete the survey.

All data from the questionnaire were downloaded from the survey administration software Google forms and saved in a Microsoft Excel 2013 file; data analysis was performed using various tools. The results were summarized by a number of respondents by region and total. The total survey results were also expressed as a percentage.

Research data was collected in accordance with Regulation (EU) 2016/679 of the European Parliament and of the Council of 27 April 2016 on the protection of natural persons with regard to the processing of personal data and on the free movement of such data [10] and Code of Professional Activity of the Latvian Association of Sociologists for Social and Market Research [11]. Participation in the survey was voluntary and by filling in the questionnaire, the respondents confirmed that they agreed to participate in the study.

## 3. Results

The characteristics of families participating in the study are given in Table 1, which showed that several generations live in one household in Latvia, as 70.7% of questionnaires indicated 10 family members; 88.9% of parents worked during the COVID-19 pandemic and in 72.4% of cases the mother was the one who prepared the lunch. In the study, a total of 6120 pupils received school catering allowance during the COVID-19 pandemic. The number of surveyed respondents covered the whole of Latvia, relatively evenly distributed by regions: Riga region—891 (17.2%), Kurzeme—497 (9.6%), Latgale—856 (16.6%), Vidzeme—694 (13.4%) and Zemgale—1051 (20.3%) taking the dispersion of the population into account. The capital of Latvia—Riga (1177 respondents—22.8%) was singled out because it has the highest number of inhabitants and schools. The obtained data allowed us to assess possible differences by region.

Ninety-four-point-five percent of all surveyed parents received a school catering allowance to provide lunch for a pupil during the pandemic (Table 2). In general, pupils received school catering allowance when they started remotely learning in March 2020 until June and from September 2020 until June 2021. The form of support in 83.7% of cases was food packs.

School catering allowance changed in 38.9% of cases, thus altering the type and frequency of support; 4324 respondents, who had noted that food packs were received as school catering allowance during the pandemic, took part in the assessment of food packs (Table 3). The distribution of respondents by regions was as follows: Riga—1103 (25.5%), Riga region—832 (19.2%), Kurzeme—342 (7.9%), Latgale—709 (16.4%), Vidzeme—589 (13.6%) and Zemgale—749 (17.3%).

Fifty-one-point-nine percent of parents stated that the received food packs should be assessed as a healthy diet, 80.6% indicated that the food packs contained both fruits and vegetables, and 71% of respondents confirmed that the food products in the packs were included in the diet of the whole family; 74.4% of respondents evaluated the food packs positively.

Analyzing the respondents’ assessment of the compliance of the food pack with a healthy diet by regions, the largest percentage of parents confirmed it in Vidzeme—62.0%, followed by Latgale—58.8%, Kurzeme—54.4%, Riga—51.1%, Zemgale—46.2%, and the lowest percentage of respondents confirmed it in the Riga region—44.1%.

**Figure 1:**
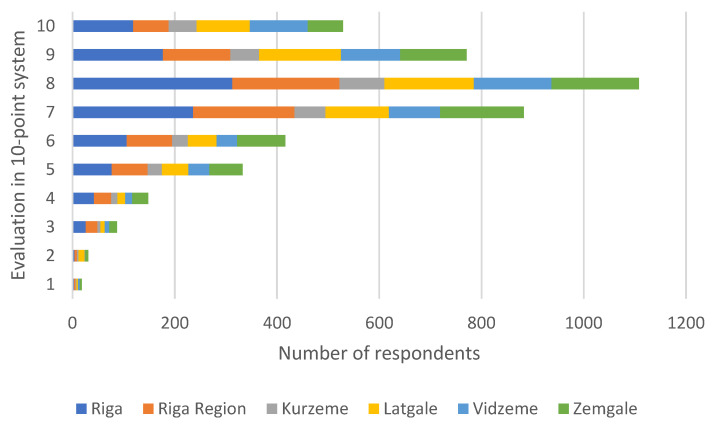
Parental evaluation of food packs in a line scale (10-point system) by regions of Latvia.

In the final stage of food pack evaluation, parents were asked to rate the composition of food packs in a line scale (10-point system), where 1 is very poor, 2—poor, 3—unsatisfactory, 4—almost satisfactory, 5—satisfactory, 6—almost good, 7—good, 8—very good, 9—excellent, 10—with distinction (Figure 1).

Overall, 76% of parents rated food packs with 7 (good) and above. The largest percentage of respondents, comprising one-quarter of all respondents (1108), rated food packs with 8 (very good). Analyzing by regions, the largest percentage of respondents who rated food packs with 7 and above was in Vidzeme—81.7%, followed by Latgale—79.4%, Riga—76.6%, Kurzeme—76.0 %, Riga region—73.1 %, and the lowest percentage of respondents was in Zemgale—71.3%. In total, the assessment was similar between regions. Food packs were given less than 4 points by 3% of respondents, who could not understand why food packs did not include local products.

The obtained data allow the conclusion to be made that parents assessed the school catering allowance (food packs) implemented in Latvia as successful, albeit with some exceptions.

## 4. Discussion

This study showed that the most popular school catering allowance during the pandemic in all regions of Latvia was food packs, as they were considered to be the simplest, safest and easiest way of providing support to families with pupils. The provision of food packs was an effective tool in reducing the deterioration of eating habits of pupils, as research shows that there are social disparities between families during the COVID-19 pandemic when the income of many families fell sharply [12]. Therefore, most parents appreciated this support, expressing their happiness with the received food products, but noting that the composition of food packs varied from school to school and that some food products should be replaced with other products. Some parents indicated that once voicing their dissatisfaction with the inclusion of certain food products in food packs, improvements were made in subsequent food packs.

Some parents misunderstood the use of food packs; the aim was to provide support for a pupil to receive a warm lunch, with the meal being composed in accordance with the recommendations of the Ministry of Health, including food products from different groups, such as grain and grain products (pasta, rice, bread, etc.), protein source products (eggs, legumes, canned meat and fish), fruit and vegetables, milk and milk products (UHT milk), potatoes, fats (oil or butter). The presence of these products in food packs was also confirmed by parents in the survey. Of course, it would be necessary to evaluate the composition of offered food packs and compare them with healthy diet recommendations, because, for example, in 31% of cases white bread was included in food packs. However, it raises the question of how this should be evaluated. From a nutritional point of view, it would be better to choose another type of bread, but if we take into account the fact that there was a crisis situation in the country, a result of which was that the state provided support, then it could be seen as acceptable.

The greatest dissatisfaction on the part of the parents was with the food products chosen to ensure the required protein content. The food packs included canned meat and fish, which some parents considered unacceptable because such food is not usually present in the family diet. Parental frustration could be seen as justified, but several aspects had to be taken into account when designing food packs, such as recommended quantities of certain food groups, price, storage conditions—at room temperature, etc. [3], which did not allow other food products to be included in food packs. Due to this situation, the contribution of parents was essential in preparing a healthy and delicious meal for a pupil from the food products in the food pack, as the influence of parents on children’s eating habits is strong [13]. In addition, parents can provide a nudge, for example, to help encourage the consumption of vegetables and fruits included in food packs, as giving a nudge has been shown to have a positive effect on a child’s dietary choices [14]; 80.6% of respondents confirmed that the food packs contained both fruits and vegetables, meaning that in this case, the responsibility for pupils consuming those lies with the parents. Statistics on the eating habits of Latvian pupils show the insufficient consumption of fruit and vegetables [15].

It is positive that in 91.0% of cases, milk was included in food packs, which is an important food product in the diet of pupils. However, some parents expressed their dissatisfaction that food packs were not intended for pupils with special dietary needs, for example, lactose intolerance, celiac diseases, etc. This reaffirms the importance of parental involvement.

Despite the goal being to provide pupils with a warm lunch, food packs were only used in 25.6% of cases, however, we believe that the inclusion of the contents of food packs in the diet of the whole family should also be assessed as a positive outcome because it could serve as a good example of how to make a balanced, healthy and delicious meal from simple food products, and due to the parents’ eating habits having a strong influence on a child’s eating choices, could also help promote healthy food consumption [16]. In addition, 74.4% of respondents rated the support as positive, indicating that they welcomed the food packs received, stating that they provided additional support in a difficult situation.

## 5. Conclusions

Given the crisis situation in the country during the COVID-19 pandemic and the rapid state response to the consequences in the form of pupil lunch support, it can be considered that food packs provided sufficient support to families with pupils, who also appreciated it. Food packs created a sense of security for families, as there were parents who had lost their jobs.

## Figures and Tables

**Table 1:** Profile of respondents.

		Riga (Capital of Latvia)	Riga Region	Kurzeme	Latgale	Vidzeme	Zemgale	Total
Number of Respondents		1177	891	497	856	694	1051	5166
Number of family members	2	15	10	5	28	8	10	76 (1.8%)
	3	39	53	32	113	44	30	311 (6.0%)
	4	86	163	64	181	68	99	661 (12.8%)
	5	34	74	46	93	53	60	360 (7.0%)
	6	4	8	6	16	11	21	66 (1.3%)
	7	1	5	3	4	3	4	20 (<1.0%)
	8	1	0	2	2	2	2	9 (<1.0%)
	9	0	1	0	1	2	2	6 (<1.0%)
	10	996	577	338	417	501	822	3651 (70.7%)
	More than 10	1	0	1	1	1	0	4 (<1.0%)
	Invalid answer	0	0	0	0	1	1	2 (<1.0%)
Subsistence provider status	Working	694	527	348	617	482	723	3391 (65.6%)
	Working in absentia	181	135	27	46	54	81	524 (10.1%)
	Working semi-remotely	204	176	39	78	54	125	676 (13.1%)
	Self-employed	0	1	3	4	2	3	13 (<1.0%)
	Pension	3	0	4	4	5	3	19 (<1.0%)
	State benefit	8	4	19	14	28	22	95 (1.8%)
	Housewife	2	3	4	2	7	1	19 (<1.0%)
	Downtime allowance	37	20	13	17	15	22	124 (2.4%)
	Unemployed	44	22	38	73	45	71	293 (5.7%)
	No answer	4	3	2	0	3	0	12 (<1.0%)
A person preparing a lunch at home	Mother	845	618	368	645	498	767	3741 (72.4%)
	Father	31	20	3	12	12	25	103 (2.0%)
	Mother and father	228	197	93	150	133	182	983 (19.0%)
	Mother or father or pupil	33	32	22	24	35	46	192 (3.7%)
	Adult ^1^	38	23	11	25	15	31	143 (2.8%)
	Pupil	2	0	0	0	2	0	4 (<1.0%)
The age of the pupil who received school catering allowance	6	1	1	3	4	4	8	21 (<1.0%)
	7	158	128	53	120	96	142	697 (11.3%)
	8	165	157	91	116	107	183	819 (13.3%)
	9	160	135	69	126	92	175	757 (12.3%)
	10	146	157	74	115	102	175	769 (12.5%)
	11	146	93	56	117	80	129	621 (10.1%)
	12	159	106	63	139	94	118	679 (11.0%)
	13	134	70	60	98	87	76	525 (8.5%)
	14	130	56	50	77	78	75	466 (7.6%)
	15	98	64	42	51	55	51	361 (5.9%)
	16	44	26	28	32	29	27	186 (3.0%)
	17+	72	32	23	33	30	29	219 (3.6%)
	Invalid answer	8	3	1	10	8	6	36 (<1.0%)

^1^ An adult who is neither the mother nor father, such as a grandparent, babysitter, etc.

**Table 2:** Characteristics of the received school catering allowance by regions of Latvia.

Questions		Riga (Capital of Latvia)	Riga Region	Kurzeme	Latgale	Vidzeme	Zemgale	Total
Number of Respondents		1177	891	497	856	694	1051	5166
Received any school catering allowance	Yes	1153	848	477	827	652	927	4884 (94.5%)
	No	17	30	18	28	37	111	241 (4.7%)
	Refuse	7	13	2	1	5	13	41 (<1.0%)
Type of school catering allowance	Food packs	1103	832	341	710	589	749	4324 (88.5%)
	Meal	19	3	47	4	12	14	99 (2.0%)
	Coupons, cards, money	25	3	62	109	42	153	394 (8.1%)
	In different ways	5	7	28	6	7	14	67 (1.4%)
Changes of school catering allowance	Yes, many times	503	301	233	338	245	387	2007 (41.1%)
	No	650	547	244	489	407	540	2877 (58.9%)

**Table 3:** Parental assessment of food packs by regions of Latvia.

Questions		Riga (Capital of Latvia)	Riga Region	Kurzeme	Latgale	Vidzeme	Zemgale	Total
Number of Respondents		1103	832	342	709	589	749	4324
Changes in composition of food packs	Different products each time	154	67	54	42	135	98	550 (12.7%)
	Everything changed	249	109	33	71	97	112	671 (15.5%)
	Certain products changed	685	632	245	577	337	526	3002 (69.4%)
	No changes	15	24	10	19	20	13	101 (2.3%)
The composition of food packs corresponded to a healthy diet	Yes	564	367	186	417	365	346	2245 (51.9%)
	Partly	464	406	137	255	195	352	1809 (41.8%)
	No	61	46	15	36	25	44	227 (5.2%)
	I do not know	3	2	1	0	1	0	7 (<1.0%)
	Other answer	11	11	3	1	3	7	36 (<1.0%)
Food packs included fruits and vegetables	Only fruit	62	45	74	210	104	68	563 (13.0%)
	Only vegetables	41	45	11	32	13	8	150 (3.5%)
	Both	974	728	240	436	463	643	3484 (80.6%)
	No fruits and vegetables	26	14	17	31	9	30	127 (2.9%)
Type of bread in food packs ^1^	Wheat bread	162	227	107	232	288	331	1347 (31.2%)
	Rye bread	120	101	68	206	167	267	929 (21.5%)
	Bread with seeds, etc.	360	160	35	10	93	241	899 (20.8%)
	Cereal cakes	112	174	1	0	7	3	297 (6.9%)
	No bread	398	272	176	348	128	145	1467 (33.9%)
The source of protein in food packs ^1^	Canned meat	1059	732	104	292	355	613	3155 (73.0%)
	Canned fish	951	629	50	181	267	418	2496 (57.7%)
	Eggs	881	446	255	629	429	576	3216 (74.4%)
	Legumes	860	605	190	450	400	548	3053 (70.6%)
	Meat products (sausages)	357	480	258	626	470	329	2520 (58.3%)
	Fresh meat	0	0	3	18	7	0	28 (<1.0%)
Milk	Included	1090	819	286	628	513	601	3937 (91.0%)
Oil	At least once	631	447	198	563	449	589	2877 (66.5%)
Use of food packs	According to purpose	295	189	99	197	142	185	1107 (25.6%)
	Included in the diet of whole family	752	621	233	497	440	540	3083 (71.3%)
	Given back to others	33	15	6	7	6	16	83 (1.9%)
Assessment of food packs as support	Positive	815	624	254	516	453	557	3219 (74.4%)
	Partially positive	198	136	58	116	92	133	733 (17.0%)
	Neutral	61	49	14	51	24	34	233 (5.4%)
	Partially negative	20	16	11	17	11	18	93 (2.2%)
	Negative	9	7	5	9	9	7	46 (1.1%)

^1^ Possibility to specify several answers.

## Data Availability

Not applicable.
